# Evaluation Tools Developed for Rett Syndrome

**DOI:** 10.3390/diagnostics13101708

**Published:** 2023-05-11

**Authors:** Meir Lotan, Jenny Downs, Michelle Stahlhut, Alberto Romano

**Affiliations:** 1 Department of Physiotherapy, Ariel University, Ariel 4070000, Israel; 2 Israeli Rett Syndrome National Evaluation Team, Ramat Gan 5211401, Israel; 3 Telethon Kids Institute, Centre for Child Health Research, The University of Western Australia, Nedlands, WA 6009, Australia; 4 School of Allied Health, Curtin University, Perth, WA 6102, Australia; 5 Department of Paediatrics and Adolescent Medicine, Center for Rett Syndrome, Rigshospitalet, 2100 Copenhagen, Denmark; 6 Department of Health System Management, Ariel University, Ariel 4070000, Israel

**Keywords:** Rett syndrome, measurement scale, clinical severity, functional abilities, behavior, physical activity

## Abstract

Rett syndrome (RTT) is a complex neurodevelopmental X-linked disorder associated with severe functional impairments and multiple comorbidities. There is wide variation in the clinical presentation, and because of its unique characteristics, several evaluation tools of clinical severity, behavior, and functional motor abilities have been proposed specifically for it. This opinion paper aims to present up-to date evaluation tools which have specifically been adapted for individuals with RTT often used by the authors in their clinical and research practice and to provide the reader with essential considerations and suggestions regarding their use. Due to the rarity of Rett syndrome, we found it important to present these scales in order to improve and professionalize their clinical work. The current article will review the following evaluation tools: (a) the Rett Assessment Rating Scale; (b) the Rett Syndrome Gross Motor Scale; (c) the Rett Syndrome Functional Scale; (d) the Functional Mobility Scale—Rett Syndrome; (e) the Two-Minute Walking Test modified for Rett syndrome; (f) the Rett Syndrome Hand Function Scale; (g) the StepWatch Activity Monitor; (h) the activPAL^TM^; (i) the Modified Bouchard Activity Record; (j) the Rett Syndrome Behavioral Questionnaire; and (k) the Rett Syndrome Fear of Movement Scale. The authors recommend that service providers consider evaluation tools validated for RTT for evaluation and monitoring to guide their clinical recommendations and management. In this article, the authors suggest factors that should be considered when using these evaluation tools to assist in interpreting scores.

## 1. Introduction

Rett syndrome (RTT) is a neurodevelopmental disorder occurring in one of every 10,000 live female births in the US [1]. In about 90% of cases, the genetic origin of RTT is due to a pathogenic variant in the X-linked gene *MECP2* coding for methyl CpG-binding protein 2 [2]. The affected individuals present a regression in development around 18 months of age, deterioration in learned motor and verbal abilities, the development of repetitive hand movements, low muscle tone, difficulties with autonomic functions, and significant cognitive impairment. Common comorbidities include scoliosis, epilepsy, autonomic dysfunction, and gastrointestinal problems [3].

Many aspects of the clinical condition are linked to a specific genetic alteration. For instance, mutations such as p.Arg270*, p.Arg255*, and p.Arg168* tend to be associated with more severe symptoms, while mutations such as p.Arg133Cys, p.Arg294*, and C-terminal deletions tend to be associated with milder symptoms [4,5]. As such, the functional spectrum presented by these individuals ranges from children with milder impairments to those who present with very severe impairments.

It is a necessity that formal and informal assessments are conducted regularly by caregivers, parents, teachers, and healthcare professionals to suggest and assess clinical care and day-to-day management. Both assessment types inform the design of intervention strategies that are most suited to the goals set for each individual. Approaches that are suitable for RTT need to be considered. For example, the movement skills of many individuals with RTT are affected by apraxia. Despite the existence of many evaluation tools for apraxia conditions [6], the uniqueness of the clinical presentation of RTT and the severity of expressive communication and cognitive impairments have necessitated the development and validation of evaluation tools for this population. Formal assessments have specific roles because they can quantify the domain of interest [7] or understand natural history. Using appropriate and validated evaluation tools is a critical component of effective clinical trials [8]. With all the information and value that comes from using validated evaluation tools (examples are presented below), it is imperative that tools serve as a step towards the advancement of the individual with RTT and not only to document inabilities and disability [9]. Therefore, the application of different evaluation tools should be carried out alongside the thoughtful collaboration between healthcare professionals, family members, and caregivers, considering strengths, goals, and aspirations for living well and maintaining a good quality of life.

### Clinical Suggestions When Using the Scales Mentioned above for Individuals with RTT

Before evaluating a person with RTT, it is crucial to consider the following points:

1. Identify the evaluation goal—The assessor should have a clear idea regarding the objective of the evaluation and should choose the most appropriate and related evaluation tool. For instance, an evaluation tool that provides a picture of the individual’s skills at a specific time could be adequate for monitoring a person’s improvement (or worsening) but could be insufficient for identifying emergent skills. When the goal of the evaluation is to recognize the impact of an intervention, the assessor should decide which tool to use after identifying the intervention program and goals.
2. Familiar vs. unfamiliar situations—Individuals with RTT may not respond well to new situations and new people [10]. Therefore, when planning an evaluation, the carers of the individual should explain what is going to happen, maintaining a positive attitude toward the assessor (e.g., saying: “Tomorrow, I will introduce you to a friend of mine who will do some fun activities with you. They work at the hospital and are very friendly and I am sure you will enjoy it”). This form of introduction should commence a few days before the evaluation. In addition, the assessor should not introduce themselves as a “professional” who is there to “evaluate the girl” but as a person who wants to get to know the girl and undertake preferred activities with her. Therefore, it is recommended to provide the person with RTT enough time to acclimate to the assessor (usually a few minutes are enough), watching her preferred stimuli (toys, videos, books, etc.) together, and involving the caregivers in order to create an initial relationship that will support the subsequent evaluation.
3. Timing of assessment—Individuals with RTT have times during the day when they function better [10,11]. If possible, plan an evaluation at a time/on the day when the person with RTT is in her optimal state. When planning an evaluation, the assessor should ask at what time of day and on what day of the week would be ideal for the girl and, if possible, schedule the evaluation accordingly. Even if it is not possible to organize the meeting on the “best” day/time, the assessor should ensure that the meeting does not take place on one of her “worst” days/times (e.g., when the child is used to sleeping or after a strenuous activity, such as a rehabilitation session, etc.). Additionally, consider other influences, such as changes in medication (e.g., new antiseizure medications), changes in educational day center (e.g., new staff members), and family changes and disruptions (e.g., parental divorce, new sibling, changes in the existing residential setting) [7].
4. Evaluation organization—When the evaluation begins, it should proceed from the tasks that are easier for the girl to those more challenging that require more active effort. In this way, the girl will experience success and will then be more able to tackle complex tasks.
5. Communication—The assessor should remember to talk to the girl throughout the evaluation meeting, modulating their voice to strengthen the relationship, observing carefully, and providing instructions and feedback. Communication should be supported by sufficient non-verbal elements (e.g., pointing, facial expressions, etc.) and augmentative communication tools usually used by the girl, is so far as possible, and caregivers.
6. Ambiance—The assessor should make sure that there is a generally relaxed atmosphere before and during the assessment.
7. Use natural situations—The person may show better abilities in certain natural situations compared to their abilities during an official examination. Therefore, whenever possible, the assessor should observe the person performing a task in situations in which they are used to doing it (e.g., if the caregivers report that the girl uses her hands to touch book pages, that situation could be used to observe reaching skills).
8. Adjust the assessment according to the person’s need—Individuals with RTT may have variable functioning at different times of the day or on different days [11]. Therefore, it is suggested that the evaluation is conducted in the presence of someone who knows the girl, as they could provide information about her “usual” functional level. If the assessor finds out that the girl is able to perform a task, but this is not observable at the time of the evaluation, a strategy to facilitate the execution of the task should be implemented. These strategies include but are not limited to: (a) providing motivational factors (each girl has individual motivational factors), (b) ensuring the availability of the girl (she should be attentive), (c) providing clear instructions, (d) providing enough time to organize and perform the task (individuals with RTT have long reaction time), and (e) offering the minimum amount of help needed to complete the task and then reduce help, if possible. If, despite all the assessor’s efforts, the girl is still unable to perform the task during the meeting, the assessor should ask the caregivers if they have a recent video of the girl performing the task and, in that case, the level of ability observed in the video can be reported in the evaluation tool or report accompanied by a note saying that it was observed in a video. Moreover, if no videos are available, but the caregivers say that the girl can perform a task, the assessor should report the ability as “not possible/observed” in the evaluation tool or report, adding a note reporting that the caregivers said that she could usually do it.
9. Assess the musculoskeletal condition—The degree of muscular shortening, articular contractures, and muscular tone abnormalities may negatively affect the individual’s functional ability [7].

With these considerations in mind, the current opinion paper presents the evaluation tools mostly used by the authors in their clinical and research practice with individuals with RTT and provides essential considerations and suggestions regarding their best use. The current paper’s authors believe that the information provided here could help to construct an evidence-informed short- and long-term evaluation and intervention plan when working with this population. All presented scales were developed or validated to be used with people with RTT and include tools for:

- Clinical severity assessment: the Rett Assessment Rating Scale (RARS);
- Assessment of gross motor abilities: the Rett Syndrome Gross Motor Scale (RSGMS), the Rett Syndrome Functional Scale (ReFuS), the Functional Mobility Scale—Rett Syndrome (FMS-RS), and the Two-Minute Walking Test (2MWT) modified for RTT;
- Manual function assessment: the Rett Syndrome Hand Function Scale (RSHFS);
- Assessment of physical activity level: the Stepwatch Activity Monitor™ (SAM), the activPAL™, and the Modified Bouchard Activity Record (M-BAR);
- Behavioral assessment: Rett Syndrome Behavioral Questionnaire (RSBQ);
- Fear of movement assessment: the Rett Syndrome Fear of Movement Scale (RSFMS).

Information related to each evaluation tool’s structure, function, and certain psychometric properties are reported in Table 1.

## 2. Clinical Severity Assessment—Rett Assessment Rating Scale (RARS)

This questionnaire was developed to assess the severity of the disorder in individuals with RTT by parents or caregivers. The tool has been used in previous studies [29,30,31,32,33,34,35,36,37] and as a basis for developing other assessment tools [38,39,40]. The structure of the RARS is based on the structure of similar tools, such as the Childhood Autism Rating Scale (CARS) [41], the Gilliam Autism Rating Scale (GARS) [42], and the Asperger Syndrome Diagnostic Scale (ASDS) [43], designed to assess the presence or absence of symptom characteristics similar to those presented by individuals with RTT [44]. The composition of the items in the tools was based on RTT diagnostic criteria provided by the DSM-IV-TR [45], recent research, and the researchers’ clinical knowledge. Each item on the scale has a short explanation, allowing the rater to understand the item and how to rate it. The individual’s characteristic is rated on a scale of 7 points: 1 = within the normal limits; 2 = low prevalence of existence or mild disability; 3 = common or moderate to severe disability; and 4 = very severe disability. Intermediate values (1.5, 2.5, and 3.5) are used when the answer does not exactly match any integer value. The RARS differs from other tools mainly in its items. For instance, among the previously proposed RTT-specific severity scales, the Kerr, Pineda, and Percy scales contain 20, 15, and 10 items, respectively [46], and the Clinical Severity Score [5] includes 13 items. Moreover, the RARS allows rating each clinical item using seven levels per item, adding to the tool’s ability to detect incremental differences. After evaluating all the items, a general summary of the score is obtained, which gives the severity level for each girl as mild (below 55 points), medium (55–81 points), and acute (higher than 81 points) [12]. In addition to the general degree of severity, individual items relating to a specific assessment area (sensory, cognitive, motor, etc.) can also be summarized. The tool was validated in 220 girls with RTT and was found to be valid and reliable [14]. On the other hand, as it is a means to measure the clinical severity level, the RARS has a low sensitivity to small changes in motor and adaptive abilities and is, therefore, less useful for evaluating the success of rehabilitation goals in the short and medium term.

## 3. Assessment of Gross Motor Abilities

### 3.1. Rett Syndrome Gross Motor Scale (RSGMS)

The tool was adapted from the Gross Motor Function Measure [47,48] to be a shorter outcome assessment [49]. The scale can be applied to the individual face-to-face or by observing recorded videos of the individual performing the provided tasks [15,49]. The RSGMS assesses an individual’s sitting, standing, and walking proficiency. It measures the person’s ability to sit on the floor or a chair, both with and without a backrest, stand for varying periods of time, walk a set number of steps, perform side steps, turn 180 degrees, navigate on a slope, step over obstacles, run, transfer from sitting to standing, rise from the floor, and bend down to pick up or touch an object before returning to a standing position. Each task is rated on a four-point scale, ranging from 0 (maximum assistance required or unable to perform) to 3 (no assistance needed), and scores are assigned based on this rating. The item scores are combined to provide a total score and three subscale scores. The evaluation of the RSGMS involved 255 families of girls with RTT, participating in the Australian Rett Syndrome Database and Danish National Center for Rett. On retesting, a difference in the total score of four points is necessary to indicate a change beyond within-subject noise [15]. A correlation between the person with RTT’s gross motor abilities and age was found using this scale, where participants aged 19 years and older had significantly lower scores than younger participants. This suggests some reduction in motor abilities in individuals with RTT at older ages, probably due to longer periods of sitting and less physical activity [15]. Decreasing scores during adulthood have also been reported [50]. The RSGMS is useful as an outcome assessment in both intervention and longitudinal studies. However, the ability to sit and stand is observed for short periods of time, and walking is scored based on the ability to walk 10 steps. Therefore, although the RSGMS enables the assessment of gross motor skills, if used for evaluation alone, it could miss improvements related to motor endurance, such as the length of time a position can be maintained or the number of steps taken. Moreover, it does not assess stair climbing and descending ability, which are important activities for the daily lives of people with RTT and their families.

### 3.2. The Rett Syndrome Functional Scale (ReFuS)

This scale was developed to detect the functional ability of girls with RTT before, during, or after an intensive motor intervention. The tool is based on independent performance only. The scale is extremely sensitive to small changes, so it is suitable when the intervention period is short. In carrying out an evaluation with this tool, the task execution times without any assistance are measured. Verbal encouragement is possible, and the presentation of favorable individuals (parents/care providers) and motivational factors are recommended (it is important to list and maintain the same conditions in each test) but avoiding physical contact is required. Each item’s score ranges from 0–12 points, so the score in the tool can range from 0 to 372 points. A higher score corresponds to better functional ability. Typical scores of young individuals with RTT who can walk ranged from 130–220 (before intensive intervention) and 200–320 (after intensive intervention) were found [51]. The first eight sections measure the ability to perform static tasks (standing and knee standing). Longer performances result in higher scores (0 = unable to perform at all; 1 = performing the task within 0–10 s; 12 = performing the task for two minutes). The rest of the sections measure mobility (walking and descending and ascending the incline and stairs), and the shorter the duration of the performance for a specific task, the higher the score (performing a specific task within 10 s = 12 points; performing a specific task within two minutes = 1; not completing the task within 2 min = 0). The tool has been used in previous studies with individuals with RTT and was found to be suitable for assessing this population and being sensitive to the slightest functional changes in the context of a short intensive intervention program [16,17]. In order to be performed, the scale needs a flight of three stairs and a 2.5 m long inclined plane. The ReFuS represents an extensive and sensitive assessment of the motor skills of people with RTT. However, due to its accuracy, its administration requires a high expertise level and a significant amount of time (an hour per evaluation), challenging its use in routine clinical practice and observational studies. Moreover, the ReFuS does not consider the person’s potential performance when support or help is provided.

### 3.3. Functional Mobility Scale—Rett Syndrome (FMS-RS)

The Functional Mobility Scale (FMS) assesses the functional mobility of children with cerebral palsy and movement disorders [52,53]. It evaluates the need and type of assistance required for the individual to walk a distance of 5 m, 50 m, and 500 m. The scale ranges from a score of 1, indicating the need for a wheelchair, to 6, indicating independent walking on any surface. FMS was used in a research study on hip dysplasia involving 31 females with RTT with a mean age of 15 years and 6 months as a descriptor of functional motor abilities [54]. The scale was later adapted specifically for use with individuals with RTT, known as FMS-RS [18]. The scoring system in this version expands the one proposed for the RSGMS, focusing on the level of assistance needed to walk a distance of 5, 50, and 500 m. A score ranging from 0 (unable) to 4 (independent) is assigned to each distance walked. The FMS-RS correlates strongly with the RSGMS and Pediatric Evaluation of Disability Inventory (PEDI) motor domain in 42 girls and women with RTT [18]. The FMS-RS can provide longitudinal data on daily ambulation for clinical monitoring and represent valuable information to report when depicting the clinical picture of an individual with RTT. However, the FMS-RS’s responsiveness to change in the population with RTT remains to be established which, as of now, limits its use as an outcome measure.

### 3.4. Two-Minute Walking Test (2MWT)

The 2MWT is a functional walking test developed as an alternative to the Six-Minute Walk Test. This test has traditionally been used for individuals who have experienced a stroke or have motor and neurological impairments [55,56,57]. Recently, it has been adapted to be utilized with individuals who have RTT [18]. In the adapted version of the test, the subject walks back and forth between two cones on a 20 m track for two minutes, and the distance covered is recorded. To enhance comprehension, motivation, and effort in subjects with RTT, the 2MWT protocol has been modified. The modified version requires two assessors and one walking assistant. The first assessor is responsible for monitoring the time and providing feedback to the evaluated person walking next to her. The second assessor counts the laps, encourages, if necessary, and has access to a chair in case it is needed. The walking assistant should be familiar with the person with RTT (such as a parent, therapist, or teacher) and provide the necessary support, whether one-handed, two-handed, or trunk support, in order to help the individual maintain balance and achieve the highest speed possible. The 2MWT showed a moderate negative correlation (r = −0.48) with the RTT Clinical Severity Score (CSS) and a with the PEDI motor domain (r = 0.43) and RSGMS (r = 0.51) in a group of 27 girls and women with RTT, whose median age was 27.4 years, with an interquartile range of 15.8–39.8 years [18]. The 2MWT was recently used in a study aimed at evaluating the functional and health-related effects of an individualized uptime participation intervention involving people with RTT for 12 weeks [58]. The 2MWT is useful as an outcome measure of walking capacity in interventions or longitudinal observations targeting gross motor skills and physical activity. However, it is important to note that the 2MWT has not been validated against a maximal exercise criterion and therefore may not reflect true capacity in individuals with RTT. Moreover, the minimal important change needs to be established.

## 4. Manual Function Assessment—Rett Syndrome Hand Function Scale (RSHFS)

The loss of manual function is typical for individuals with RTT during the initial period of the disorder, and the RSHFS is a valuable tool to score the person’s level of hand functioning. The RSHFS protocol was developed based on the Hand Apraxia Scale, a 10-item measure of manual functioning [59]. The hand function of the person with RTT is scored on a discrete scale between 1 (no observed hand function) and 8 (skills for levels 7 and, when the hand is approaching an object, hand orientation and size recognition closely approximates the position and size of the object) [19]. Each skill level includes the ability to carry out the actions outlined by the previous level as well as other actions of a higher functional level. The scale evaluates the ability to grasp, pick up, and hold large (e.g., small ball or toy) and small (e.g., candy, small pieces of food) objects; the type of grasp used (raking, radial side, or scissor grasp); the ability to transfer an object from one hand to the other; and the presence of the accurate pre-shaping of the hand approaching an object. The scale can be administered directly to the individual or by observing recorded videos of the person performing the required tasks [19]. The scale was used to evaluate the hand function of 144 girls with RTT who participated in an Australian population-based study [19]. The study mentioned above found that 30% of the participants could not grasp any object, 17% held an object for more than 2 s when placed in their hand, and about 12% grabbed the object independently and then held it independently in their palm. About 40.3% could use their fingers to grasp objects, and approximately half of them (20%) could transfer objects from hand to hand [19]. Other studies suggest that the manual abilities of individuals with RTT are relatively stable over the years [60,61], with younger children being at higher risk of losing hand function than adults [62]. The RSHFS was used to evaluate the changes in the hand function of three girls with RTT participating in an 18-month training program based on conductive environment principles [17]. This scale is a valuable tool for monitoring the ability to grasp and hold an object of longitudinal design or before and after treatments and is able to capture small increments of this critical fine motor task. However, it does not evaluate reaching ability. Knowing the reaching ability of a person provides crucial information to the person’s caregivers, allowing them to alter their own interactions with the individual (e.g., place an object or their face at the correct distance, allowing the person to reach and grasp or touch it).

## 5. Assessment of Physical Activity Level

### 5.1. StepWatch Activity Monitor™ (SAM)

Step counters and pedometers are objective, inexpensive, valid, reliable, and relatively easy-to-use measures of physical activity commonly used within the normative population [63]. Moreover, walking is a commonly reported physical activity of individuals with intellectual disabilities [64,65]. The convergent validity of pedometers with other measures of physical activity, such as accelerometers, observation, and energy expenditure, has been repeatedly tested and established, proving the validity of these tools [66]. The specific measuring device validated for individuals with RTT is the StepWatch Activity Monitor™ (SAM–Modus Health, LLC, Washington, DC, USA), an accelerometer-based device that attaches to the ankle using a Velcro strap, which responds to acceleration, position, and timing. It measures the number of steps taken [21]. This measure was previously validated for individuals with intellectual and developmental disabilities [67]. The use of SAM for individuals with RTT was validated through 64 participants (mean age 17 years 7 months; SD± 9 years) and compared with the activPAL™ (PAL Technologies, Glasgow, UK) and ActiGraph (ActiGraph, Pensacola, FL, USA) devices. The following criteria were used: each participant contributed a minimum of four days of data, including at least one weekend day and a minimum of 100 steps per day and a minimum of nine hours of daily wear time was recorded. Device data were verified against diary data filled in by direct care providers. The findings suggested that the median daily step count was 5093, with adults taking fewer steps and being more sedentary [68]. In a different project, 20 individuals with RTT who could walk with or without external support walked for 20–30 min while wearing the device. The participants were filmed, and two independent observers viewed the videos to corroborate the number of steps. The number of steps observed on the videotapes was the criterion measure. Bland–Altman analyses [69] were conducted to determine agreement (i.e., accuracy) between the average step count derived from the videotape and recorded by the device each minute. The videotape analysis revealed that 20 participants took the same number of steps in at least two one-minute epochs. The reliability of the step count pairs measured by the SAM was more accurate than the measurement with the activPAL™ and ActiGraph devices. The standard measurement error was six steps/min, and the minimal detectable difference was 17 steps/min. Step counts measured via the SAM also demonstrated good repeatability in the population with RTT [68]. Following this validation process, this measure was successfully used in other research projects [18,70,71]. Access to the software and technical skills to download and interpret data are needed. Moreover, the caregiver must remember to place the device on the person with RTT during waking hours for a minimum of 4 days over a one-week measurement period in order to yield valid data.

### 5.2. activPAL™

The activPAL™ device uses a uniaxial accelerometer and inclinometer to determine step count and the time spent in lying down/sitting as well as standing and walking (uptime) positions. The device is attached to the person’s thigh using adhesive pads and provides data measuring the thigh inclination and acceleration [21]. In a recent study, 26 individuals with RTT wearing the activPAL™ were video-recorded during activities performed while sitting, standing, and walking [22]. On the other hand, the authors reported that the activPAL™ overestimated the duration of the standing position and underestimated the duration of walking. This discrepancy was related to the previously reported difficulty of the activPAL™ in accurately recording walking at a slow pace [21]. Following these results, the activPAL™ was established as the most accurate device to estimate the sedentary time of people with RTT and was subsequently used to describe the patterns of sedentary time in this population [72]. In this study, the sedentary time of 48 people with RTT (median age 22.0 years; range 5.5–60.5 years) was monitored for seven consecutive days using the activPAL™ and analyzed. The results showed a mean sedentary time corresponding to 83.3% (SD 13.9%) of waking hours and that older participants (above 33.5 years) and those with higher disease severity were more sedentary than those younger or with milder RTT presentation. This information highlights the need for accurate measurement of sedentary time in RTT, which can be effectively achieved using the activPAL™ device. It has been used to measure sedentary time in intervention studies [58,71]. On the other hand, the activPAL™ was found to underestimate the step count of people with RTT, especially when walking at a slow pace. Whilst it can be used to measure sedentary behaviors, it does not accurately measure walking activity when reflected as steps [21].

### 5.3. Modified Bouchard Activity Record (M-BAR)

The BAR is a whole-day diary card in which every 15 min of the day a number can be assigned between one and nine to represent either sedentary behavior or a particular level of physical activity intensity [73]. When validating the scale for RTT, the number of categories was reduced from nine to five to modify the scale and make it more relevant to people with this disability. The modified scale comprises the following categories: “lying”, “sitting for mealtime or other activities”, “light activities in standing”, “walking at a slow intensity”, and “walking at a vigorous intensity”. “Walking at a slow intensity” was defined as “walking gently, such as when walking around the house”. “Walking at a vigorous intensity” was defined as “fast or more vigorous than gentle walking”. Higher scores correspond to higher physical activity levels [23]. The reports on the M-BAR by parents were compared with the reading of the SAM and activPAL™ for active (walking) and passive (lying down and sitting) periods of time, respectively [22,23]. According to M-BAR data, those who needed assistance with walking spent significantly more time sitting (9 h 15 min vs. 6 h 15 min—*p* < 0.001) and less time standing (1 h vs. 2 h 15 min—*p* = 0.04) than those who walked independently. The M-BAR was validated with 43 individuals with RTT (mean age: 21 years; SD 9 years) to evaluate the “uptime” in this population [23]. Moreover, a further analysis conducted by Stahlhut et al. [22] demonstrated a strong association between the sedentary time of people with RTT measured with the M-BAR and activPAL™. The authors reported that each additional minute of sedentary time recorded with the ActivPAL™ was associated with nearly one additional minute of sedentary time as classified on the M-BAR. These results support the use of the M-BAR as a simple proxy-report assessment of physical activity in individuals with RTT. Data should be interpreted with some caution because self-reported physical activity is vulnerable to over-estimation compared with objective measures [74].

## 6. Behavioral Assessment—Rett Syndrome Behavioral Questionnaire (RSBQ)

Some behavioral features have been associated with RTT [75,76]. Although hand stereotypies, hyperventilation, and breath-holding are the most commonly described, Mount et al. [77] reported other frequently observed behaviors, such as indifference to people, poor eye contact, sleep abnormalities, anxiety features, mood changes, and others. As specific behaviors were found in the past to relate to specific genetically oriented disorders [78], Mount et al. [25] aimed to measure the specific behavioral phenotype associated with RTT to distinguish it from other female individuals with an intellectual disability that was not RTT. The authors developed a checklist of typical RTT behavioral and emotional characteristics to form the RSBQ. To develop the RSBQ, the Developmental Behavior Checklist (DBC) [79], a well-validated measure of behavioral disorders in individuals with intellectual disabilities, was considered, and RTT-specific behavioral and emotional features were identified by reviewing the existing literature. Each item is represented by a sentence that is scored by the person’s caregiver on a three-point Likert scale, according to how well the item describes the individual’s behavior (0 = the proposed behavior does not describe the person; 1 = the proposed behavior sometimes describes the person; and 2 = the proposed behavior often describes the person). Data for the validation of this questionnaire were achieved by assessing the behavior of young (under 19 years of age) girls with RTT (N= 143) against data from girls with severe to profound intellectual disability (N = 85). Direct caregiver’s reports provided the data. The RSBQ has been used as an outcome in recent clinical trials, evaluating the effects of Mecasermin [80] and Trofinetide [81,82] for RTT. The wide range of behavioral manifestations shown by people with RTT should be taken into consideration by healthcare providers when planning individualized therapeutic interventions for this population. Although useful for obtaining a behavioral picture of a person with RTT within the clinical practice, the validity of the RSBQ in evaluating the clinical trial effects was questioned in a recent critical analysis by Hou and colleagues [83]. The authors evaluated and highlighted the lack of the RSBQ’s scores reproducibility and its inadequacy as a clinical trial outcome measure. As a consequence, the authors remarked that the RSBQ was first proposed to distinguish individuals with RTT from individuals with other profound intellectual disabilities based on behavioral characteristics and its validity for other scopes (such as assessing the phenotypic severity of RTT subjects, stratifying eligibility, and measuring outcomes in clinical trials) is not justified by the available validation processes. This conclusion from Hou et al. [27] was criticized in a subsequent correspondence from Oberman et al. [83], but the need for the further validation of the RSBQ was acknowledged (for further details on the correspondence also see [84]). Therefore, an additional factor analysis of the RSBQ was recently conducted using 323 pediatric datasets and 309 adult datasets collected from families in the USA, the UK, Denmark, and Australia [24]. Analyses yielded six meaningful pediatric subscales and seven meaningful subscales for adults. These new factor analyses generally replicated the original factor structure. Total score, total subscale scores (excluding dropped items), and a modified General Mood Subscale showed good psychometric properties. These new data support the notion that the original subscales of the RSBQ provide important information, but the stronger psychometric properties of the novel factor structure suggest that this alternative scoring could be more suitable for research and clinical practice. As a final note, when asking parents to fill in the RSBQ, it should be remembered that some items use vague language such as “there are times when” and “for no apparent reason” that parents could find problematic. Finally, items about walking and standing are not filled in if the person cannot perform such activities. This choice leads non-ambulatory children to be scored as being lower severity.

## 7. Fear of Movement Assessment—Rett Syndrome Fear of Movement Scale (RSFMS) 

One of the behavioral characteristics reported in girls with RTT is fear of movement (FOM) [25]. The consequences of FOM may be a significant barrier to the acquisition of functional movement and ambulation in girls with RTT and may cause the development of secondary limitations [85,86]. The assessment of FOM makes it possible to assess the intensity and severity of FOM in RTT. Such an evaluation will enable the person with RTT to be presented with an appropriate intervention mode. Interventions that reduce fear of movement have been found to improve the quality of life of the person with RTT and their family, enhancing their involvement in activities that include movement [31,32]. The scoring of the RSFMS system is the same as the RSBQ, which was used as a basis for its development. The initial validation of the RSFMS was conducted with 25 females (ages 5–33 years), including a group of 12 girls with RTT and a control group of 13 typically developed girls of equivalent ages [28]. Even though the RSFMS was found to have high psychometric value, the full validation of the scale is required for further clinical use. The authors suggest that using the scale will help clinicians working with individuals with RTT in planning appropriate management strategies for this group of individuals [28], thereby reducing the FOM, which might restrict their freedom to experience and explore their physical surroundings. The RSFMS is a recently proposed tool with preliminary validation data only. Therefore, even though it can be helpful in clinical practice to identify the presence of FOM, it should be used with caution in research studies. Moreover, it could be revised following further analyses and broader use. Items related to walking and standing are not filled in if the person cannot perform such activities. The authors do not think this issue will lead ambulatory children to receive higher scores (related to greater severity), as these children do not present a fear of movement in the sitting position. This issue should be further assessed in future research.

## 8. Future Developments

The current opinion paper is focused on evaluation tools dedicated to motor function due to the authors’ experience in this field. Although the existing evaluation tools allow for the assessment of several aspects of RTT, a few of these remain unexplored to their full extent. For instance, objectively scoring this population’s reaching ability is needed. As discussed above, every evaluation tool presents advantages and disadvantages and could be adequate for one specific aim but not another. Therefore, future research projects should focus on completing validation processes for each scale mentioned above. Furthermore, there are physiological measures that have been used in the general population, such as heart rate and respiration measurements, using technological gadgets. The possibility of using such devices for individuals with RTT has been questioned in previous research projects [87]. The validity of such devices for individuals with RTT should be examined thoroughly in the future.

## 9. Summary

As individuals with RTT present functional diversity in different areas, validated tools are recommended to assess their abilities as a group as well as before, during, and after treatments. The current article has presented evaluation tools built, modified, and validated specifically for individuals with RTT. These evaluation tools stand alone or can be used together to enable a comprehensive and appropriate assessment of an individual’s function according to a specific research protocol or an intervention program. The proposed evaluation tools cover the evaluation of RTT severity level (RARS), level of gross motor skills (RSGMS), independently performable motor skills (ReFuS), global mobility (FMS and 2MWT), purposeful manual functioning (RSHFS), physical activity (SAM™, activPAL™, and M-BAR), and fear of movement (RSFMS). The authors hope that by presenting the evaluation tools described above, better therapeutic assessments will be implemented by clinicians working with individuals with RTT, enabling finer therapeutic goal setting and better outcome evaluation during clinical interventions and research procedures. Moreover, the authors suggested options for the future development of evaluation tools for RTT and some futuristic research directions.

## Figures and Tables

**Table 1:** Structure and psychometric properties of the presented evaluation tools.

Evaluation Tool Acronym	Reference Article	N° of Items	What It Measures	Subsections/Subscales	Available Psychometric Characteristics
RARS	[12]	31	RTT severity	- Cognitive; - Sensory; - Motor; - Emotional; - Autonomic; - Other typical characteristics and behaviors	Internal consistency (Cronbach’s Alpha): - total score: α = 0.91; - subsection scores: α = 0.81–0.93 [13,14].
RSGMS	[15]	15	Support needed for gross motor abilities	- Sitting; - Standing and walking; - Challenge	Total score internal consistency: Cronbach’s Alpha = 0.96.Total score test–retest reliability: ICC = 0.99. Known group validities: lower scores in older age groups and people with mutations associated with greater clinical severity. Minimal detectable difference: 4 points (total possible score 45 points) [15].
ReFuS	[16]	31	Independent gross motor abilities	- Performance duration; - Time to complete	Inter-rater reliability: ICC = 0.99 [17].
FMS-RS	[18]	3	Level of assistance needed to walk a 5-, 50-, and 500 m distance		Test–retest reliability for each distance: ICC > 0.90 [18].
2MWT	[18]		Walking capacity		Test–retest reliability: ICC > 0.85.Minimal detectable difference: 38 m [18].
RSHFS	[19]		Purposeful hand function (grasping objects)		Known group validities: expected differences by type of pathogenetic mutation: compared to C-terminal deletion, range of OR 0.19 (95% CI 0.04–0.95)–2.65 (95% CI 0.57–12.26) [19]. Inter-rater reliability: mean (95% CI) weighted Kappa value above 0.5 and Fleiss Kappa = 0.52 [20].
SAM	[21]		Number of steps		Agreement (against videotape-observed step counts): mean difference (limit of agreement) = −1 (16) steps/min. Repeatability (steps in two one-minute epochs): ICC = 0.91; 95% CI = 0.79–0.96. Minimal detectable difference: 17 steps/min [21].
activePAL	[21]		Time spent lying down/sitting, standing, and walking		Agreement (with videotape-observed activity duration: mean difference (limit of agreement)): - lying down/sitting: –1.0 (6.3) minutes; - standing: 2.5 (7.0) minutes; - walking: –1.6 (5.2) minutes. Consistency (between duration of observed and recorded activity): lying down/sitting: ICC = 0.996, 95% CI = 0.993–0.998; standing: ICC = 0.979; 95% CI = 0.928–0.992; walking: ICC = 0.919; 95% CI = 0.782–0.966 [22].
M-BAR	[23]		Parent-reported level of physical activity and uptime		Construct validity (“uptime” against the daily number of steps): - r^2^ = 0.58; *p* < 0.001 for those able to walk independently; - r^2^ = 0.60; *p* < 0.001 for those able to walk, with or without help [23].
RSBQ(currently under revision [24])	[25]	45	RTT-specific behavioral and emotional manifestations	- General mood; - Breathing problems; - Hand behavior; - Face movements; - Body rocking and expressionless face; - Night-time behaviors; - Fear/anxiety; Walking/standing	Test–retest reliability: Cronbach’s Alpha ≥ 0.82. Internal consistency: Cronbach’s Alpha ≥ 0.88 [26,27].
RSFMS	[28]	36	Fear of movement		Inter-rater reliability: r = 0.993; *p* < 0.001. Intra-rater: r = 0.958; *p* < 0.001. Internal consistency: Cronbach’s alpha = 0.765. Accuracy = 85.5–94.4% [28].

Abbreviation list: ICC = intraclass correlation coefficient; CI = confidence interval.

## Data Availability

Data sharing not applicable.
